# Highly Charged Cellulose Nanocrystals Applied as A Water Treatment Flocculant

**DOI:** 10.3390/nano9020272

**Published:** 2019-02-15

**Authors:** Dana Morantes, Efrén Muñoz, Doron Kam, Oded Shoseyov

**Affiliations:** 1 Development & Application of New Materials (DANUM), Faculty of Sciences Postgraduate, Chemistry Master Program, Universidad Pedagógica y Tecnológica de Colombia (UPTC), Tunja 150001, Colombia; dana.morantes@uptc.edu.co (D.M.); efren17@gmail.com (E.M.); 2 Robert H. Smith Faculty of Agriculture, Food and Environment and The Harvey M. Krueger Family Center for Nanoscience and Nanotechnology, The Hebrew University of Jerusalem, Rehovot 76100, Israel; doron.kam@mail.huji.ac.il

**Keywords:** nanocellulose, surface modification, quaternary agent, flocculation

## Abstract

Various cellulosic materials have replaced petroleum-derived polymers, offering natural and sustainable alternatives. Among them, cellulose nanocrystals (CNC) feature an easily modifiable surface, enabling the exploration of a wide spectrum of applications. In this work, the quaternary agent 3-chloro-2-hydroxypropyltrimethylammonium chloride (CHPTAC) was used as a cationic graft on CNCs, to form a novel water treatment flocculant. The resulting material was chemically and structurally characterized by the determination of Zeta potential; degree of substitution by elemental analysis; hydrodynamic size by dynamic light scattering (DLS) and infrared spectroscopy with Fourier Transform Infrared (FT-IR); and X-ray diffraction (XRD). The flocculation capacity of cationic cellulose nanocrystals (CNC-EPTMAC) was evaluated in a jar test filled with an 0.25 wt.% silica (SiO2) suspension. CNC-EPTMAC proved to be an effective water treatment flocculant, reducing turbidity by up to 99.7% at a concentration of only 2 ppm. This work demonstrates a natural and environmentally sustainable alternative to homologous commercial flocculants.

## 1. Introduction

Water clarity is important in products destined for human consumption and in industrial processes that require the usage of water. Water turbidity is caused by suspended and colloidal matter such as clay, finely divided organic and inorganic matter and microscopic organisms [1]. Flocculation is an essential process in water treatment, as it plays a key role in solid–liquid separation by the aggregation of colloidal particles; therefore, it is commonly used to reduce turbidity in waters. 

Metallic salts or polymers are used to induce flocculation, but show low solubility [2]. When dissolved in water, metallic salts form cationic species, which are absorbed by negatively charged dirt particles. Polymers are classified as natural coagulants (e.g., starch, guar gum and sodium alginate) or synthetic flocculants (e.g., ionic polymeric flocculents) [3] and are primarily characterized by high molecular weight, concentration and ionic charge, which all contribute to their polymer flocculation capacities [4]. However, their disadvantages include higher costs and the remnants they leave in water, which fail to align with the increasing demand for environmentally friendly reagents and technologies [5]. Natural polymers have been extensively researched with the goal of replacing inorganic and synthetic polymers [6]. Use of organic or biodegradable polymers brings advantages, including flocs with high shear strength, stronger elastic bonding resulting from interparticle binding, and reduced sensitivity to pH variations, which enables the treatment of a broader variety of water types [7,8]. Quarternized polymeric flocculants based on ammonia were developed in the 1950s and gained rapid acceptance and are now some of the most widely used reagents [9]. Since most natural colloid particles and many dyes in waste water are negatively charged, cationic polysaccharides are of particular interest as potential flocculants. A polysaccharide cation derived from a reaction with a quaternary ammonium has been shown to be an effective flocculant in wastewater over a wide pH range [10]. 

In recent years, cellulose nanocrystals (CNCs) have attracted significant attention due to their renewable and biodegradable properties [11], alongside other properties such as their low density [12], high tensile strength [13] and high surface area [14]. The presence of hydroxyl groups on the CNC surface enables simple modifications aimed at altering its hydrophilicity by introducing a desired functionality, thereby targeting the CNC for a specific application [15].

Ionic CNCs may be very efficient flocculating agents as they are high aspect ratio rigid particles. In addition, their very large external surface area can be modified to create active sites for flocculation, which would allow for floc formation at low concentrations [16]. Grafting CNC with 4-vinylpyridine resulted in pH-responsive reversible flocculants, a useful property for biomedical applications [17]. Another straightforward modification of CNC was introduced by Hasani et al., who cationized CNC with epoxy-propyltrimethylammonium chloride (EPTMAC), which resulted in a electrostatic stable dispersion [18]. The reaction was further characterized in a molecular composition in solution-state NMR [19]. 

In this paper, we evaluate the flocculation capacity of cationic CNCs in water treatment. Cationization of the surface of sulfuric acid-hydrolyzed CNCs was achieved with EPTMAC, resulting from 3-chloro-2-hydroxypropyltrimethylammonium chloride (CHPTAC) hydrolysis in basic medium, yielding cationic CNC-EPTMAC. The chemical composition, surface charge, dimensions, and crystalline properties were examined; and the flocculant capacity, as measured by turbidity reduction in a jar test, was assessed.

## 2. Materials and Methods 

### 2.1. Materials 

Aqueous CNC was supplied by Melodea Ltd. (Rehovot, Israel). Hidroxypropylmethylammonium chloride (CHPTAC) and sodium hydroxide (NaOH), which were of analytical grade, and silicon dioxide (SiO_2_) nanopowder (spherical and porous, 5-15 nm particle size, 99.5% trace metals basis) were purchased from Sigma–Aldrich (Rehovot, Israel). The commercial flocculants Magnafloc LT22s and Magnafloc LT 22s-DWI (BASF, Ludwigshafen, Germany) were used for the flocculation test. 

### 2.2. Preparation of Cationic CNCs 

NaOH solution was added to a CNC dispersion (2 wt.%) to obtain a CNC concentration of 2M. The solution was stirred for 30 min at room temperature. Then, different molar ratios of CHPTAC (6, 9 or 12) per unit of anhydroglucose were added and stirred at 25 °C for different periods of time (4, 8 or 24 h). The reaction product was purified by dialysis (12-14 kDa dialysis bag) in distilled water (DW) over 3 days, with the water being replaced three times each day. The resulting cellulose nanocrystals—epoxypropyl trimethyl ammonium (CNC-EPTMAC) was dried by film casting for the purpose of characterization analysis. 

### 2.3. Zeta Potential

The Zeta potential of CNCs was measured before and after modification, using the Zetasizer Nano-ZS platform (Malvern, UK). More specifically, 0.1% (wt.%) samples were inserted into capillary cells (DTS1070); measurements were performed at 25 °C. Unmodified CNC samples were tested in the presence of 5 mM NaCl and CNC-EPTMAC was tested free of salt. Data analysis was performed using ZetaSizer software which converts mobility (μ) to Zeta potential using the Smoluchwski approximation; the data reported are the average of three measurements. pH was modified manually.

### 2.4. Degree of Substitution

The percentage of nitrogen was determined using the Thermo EA 1120 equipment for C H, N, S and O elemental analysis of solid samples. For this analysis, dried CNC-EPTMAC film samples (1–2 mg) were inserted into a tin capsule; the tin capsule was used as a blank control. The relation between the nitrogen content and degree of substitution (DS) of the anhydroglucose unit (AGU) is given by Equation (1) and relates to the number of quaternary ammonium groups.

(1) $$\mathrm{DS}=\;\frac{162\;\times \;\%N}{14-\;282.31\;\times \;\%N}$$

where 14, 162 and 282.31 correspond to the molecular weight of nitrogen, AGU and CNC-EPTMAC, respectively. %N is the percentage of nitrogen.

AGU per CNC was calculated by assuming that one unit cell (1.08 nm) contained four AGUs. Moreover, we assumed that the CNC particle is a square prism and calculated only the AGU on the surface of the prism particle (= 4 × length × diameter), disregarding CNC particle ends that are assumed to be non-reactive.

### 2.5. Dynamic Light Scattering

Hydrodynamic size measurements and polydispersity index (PdI) were determined by dynamic light scattering (DLS), measured with the Zetasizer Nano ZS equipment (Malvern, UK). Suspensions were diluted in DW to 0.01 wt.% and measurements were performed at 25 °C, in disposable polystyrene cuvettes.

### 2.6. Fourier-Transform Infrared and X-ray Diffraction 

Fourier-transform infrared (FTIR) assessments were performed with NICOLET 6700, using the attenuated total reflectance (ATR) technique on dried CNC-EPTMAC film samples. Measurements were acquired by an average of 32 sweeps between 550–4000 cm^−1^ and a resolution of 4 cm^−1^. Structural analysis was performed using X-ray diffraction (XRD) (Bruker AXS D8 Advance Diffractometer, Karlsruhe, Germany) at a scanning rate of 5 °C per min and using Cu Kα as a radiation source (λ = 1.54060 Å), operating at 40 kV and 30 mA. The XRD patterns were obtained over the angular range of 2*θ* = 10–50°. The relative crystallinity indices (RCIs) were calculated by Equation (2):

(2) $$RCIs\;=\;\frac{I_{200}\;-\;I_{min}}{I_{200}}*100\%$$

where *I*_200_ is the intensity of the (200) reflection plane above baseline and *I*_min_ is the minimum intensity above baseline near 2*θ* = 18°, corresponding to the minimum between the planes (200) and (110) in the diffractogram [20].

### 2.7. Thermal Properties

Thermal properties were assessed by thermogravimetric analysis (TGA) and differential scanning calorimetry (DSC), performed using the thermal analyzer Labsys evo DTA/DSC (Setaram, Caluire, France). Approximately 18.8 mg CNCs dried films and 14.6 mg dried CNC-EPTMAC films were subjected to a heating ramp from 20 °C to 450 °C, at 10 °C increments and a nitrogen flow of 50 mL per minute. A differential thermal analysis (DTA) graph was plotted from the derivative of the TGA data obtained. 

### 2.8. Flocculation Test 

Flocculation potential was measured by turbidity reduction in a suspension of silica (SiO_2_, 0.25% wt.%), simulating wastewater, which was quantified by nephelometry and measured in nephelometric turbidity units (NTU). CNC-EPTMAC was added as a flocculant at different concentrations in a range of 0.25–10 ppm. The sample was then agitated by rapid stirring (200 rpm, 2 min) and slow stirring (80 rpm, 5 min) and finally allowed to sediment (10 min) [21]. Flocculus formation was visually assessed and the turbidity of the supernatant was measured using an HACH Model 2100N turbidimeter. As a control, the same procedure was performed with CNC as a flocculant and with commercial flocculants (Magnafloc LT 22s and Magnafloc LT 22s-DWI). 

## 3. Results

### 3.1. Synthesis of Cationic CNC 

**Figure 1:**
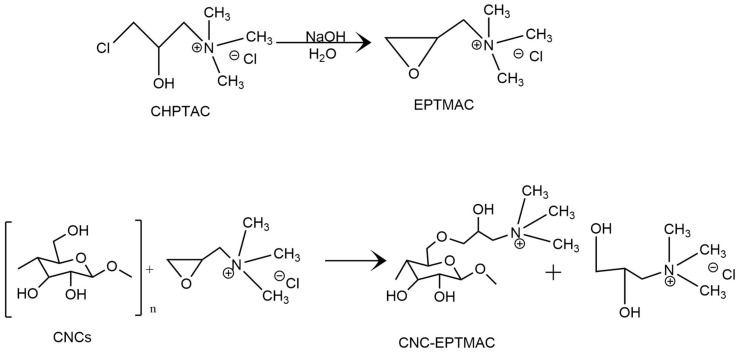
CNC-EPTMAC etherification reaction.

CNC etherification was achieved via alkaline hydrolysis. A basic medium was required for the activation of the hydroxyl groups as well as for cleavage of the sulfate half-ester groups in order to ensure the pure cationic nature, at the same time CHPTAC formed the reactive epoxy functional group EPTMAC [22]. As a result, a nucleophilic reaction between the alkali group (-OH) of the CNCs and the EPTMAC epoxy group occurred, yielding CNC-EPTMAC (Figure 1). 

Various reaction conditions, i.e., different molar ratios (6, 9, and 12) of CHPTAC by AGU and reaction times (4, 8, and 12 h) were evaluated. CNC etherification with CHPTAC resulted in a suspension with thixotropic gelation properties, likely due to the high viscosity of the suspensions [23]. Generally, CNCs prepared via sulfuric acid hydrolysis possess a negative surface charge due to the sulfate-half ester groups, which results in an electrostatically stable colloidal system and prevents the sedimentation and agglomeration of CNC in aqueous suspensions [24]. After etherification, the suspension separated into two phases: a clear phase and gel phase. The gel was the result of bundle formation, which depends on the degree of electrostatic repulsion or steric hindrance between the CNC’s particles. The higher the surface charge, the higher the electrostatic repulsion and the lower the bundle size. The bundles can be broken through sonication, which re-solubilizes the CNC-EPTMAC, weakening the formation of hydrophobic flocs [25].

Experimental observations suggest that the order of reactivity of the hydroxyl groups of the CNCs as nucleophiles was C6 = C2 > C3, favoring C6, the most reactive carbon [15]. The etherification reaction seeks to create properties that are more compatible with polar matrices, mainly solubility in water [26].

At the same time, EPTMAC is consumed in the competition between two reactions: The first being the cationization of CNCs, which is desirable, and the second being the hydrolysis of EPTMAC, leading to the formation of undesirable diols, which occurs in cases of high water content in the reaction system [27]. The higher the water content, the more hydrolysis of EPTMAC will be favored, resulting in less EPTMAC available for the cationization reaction and ultimately to a decrease in the efficiency of the reaction system. Therefore, the water content of the system is critical for the cationization process [28]. Moreover, some of the non-CNC reactive EPTMAC may be hydrolyzed to give a quaternary ammonium diol which may form a stable hydrogen bond that may not be easily removed by dialysis. We cannot rule out the possibility that at least some of the quaternary ammonium groups are not covalently bound to the CNC.

### 3.2. Zeta Potential, Degree of Substitution and Hydrodynamic Size 

As the molar ratio of the cationic agent increased, the Zeta potential, the principle optimization variable of the reaction, increased; the Zeta potential got closer and closer to zero until it reached a positive value (Table 1). In contrast, the reaction time did not have a significant impact on Zeta potential variations. In summary, the most favorable conditions for the reaction in terms of Zeta potential were with a molar ratio of 12 mol of CHPTAC per AGU and a 4-h reaction, yielding a Zeta potential of + 40.4 ± 0.7 mV and a yield of 97.5%. Grafting of cationic charges on the modification surface was demonstrated by the negative potential corresponding to the sulfate half ester groups on the surface of CNCs, resulting from the sulfuric acid reaction, which influenced the electrostatic stability of the aqueous suspension of CNCs [14,29]. These observations are in agreement with previous characterizations of interactions between quaternary ammonium surfactants and cellulose [30], cellulose nanofibers [26] and CNCs [18,28,31]. In electrostatic stabilization of colloids, the intensity of Zeta potential implies stability, where values above 30 mV or below −30 mV are generally considered stable [32]. 

EPTMAC grafting was confirmed through elemental analysis (N, C, O, S, and H), where the percentage of nitrogen corresponds to the ammonium group content. Natural cellulose molecules do not contain nitrogen and, therefore, any trace of nitrogen in CNCs modified with EPTMAC is an indication that cationic group grafts are present on the surface of the CNCs. As expected, the unmodified CNCs did not contain nitrogen, while those modified with EPTMAC showed significant amounts of nitrogen, which were proportional to the EPTMAC concentration. The CNC cationization reaction yielded 1.43% nitrogen for a 12 mol of CHPTAC per AGU and 4-h reaction, in agreement with Kaboorani and Riedl et al., who reported on 1.23% nitrogen with a concentration of 1.4 mmol/g CHPTAC and a 4 h reaction on CNCs [33]. DS increased as a function of reaction time, however, it did not vary significantly between molar ratios of 9 and 12.

When replacing the more reactive hydroxyl group (-OH) of carbon 6, it would be expected that the maximum DS for this reaction would be 1. Our optimal product obtained a DS of 0.23, following a 4-h reaction, which demonstrated that the reaction time does not influence DS. Namely, the reaction required less energy expenditure to obtain a high cationic content. Another published work reported on a DS of 0.02 per AGU^−1^ for cotton-derived nanocrystals and 0.04 per AGU^−1^ for wood-derived nanocrystals [19]. 

The hydrodynamic size of CNC-EPTMAC, measured by DLS, revealed a stable particle size with a low PDI, with no direct influence of molar ratio and reaction time on the size. However, the size of the modified rods was greater compared to the unmodified CNCs. This is due to the fact that cation-modified CNCs prepared in a high-water-content reaction system have a very low surface charge. Therefore, due to the weak electrostatic repulsion, cationically modified CNCs may agglomerate and form larger bundles [28].

### 3.3. pH Stability

**Figure 2:**
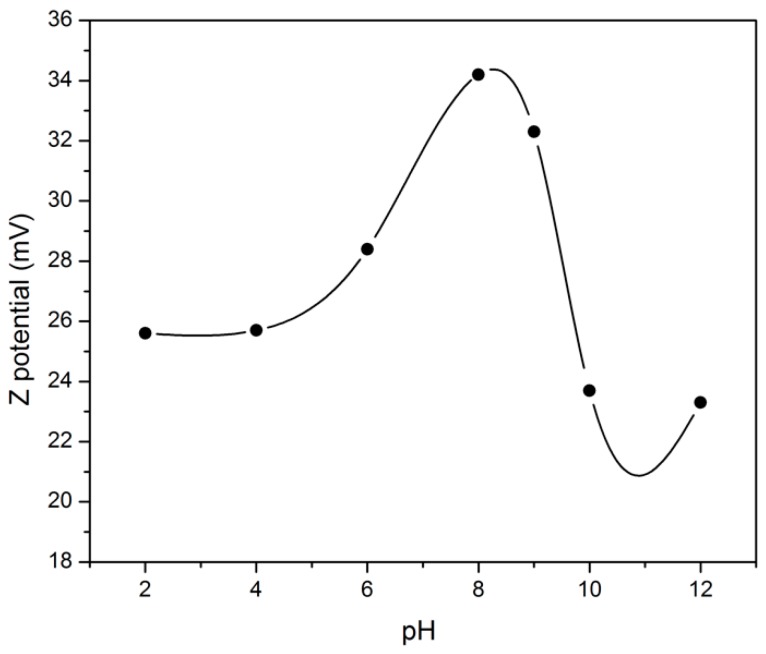
Zeta potential measurements of CNC-EPTMAC.

The stability of the pH of the aqueous medium is one of the most important factors of water treatment and can reduce the costs of pre-treatment pH adjustments [34]. In a colloidal system that includes the suspension medium and CNC-EPTMAC particles, features like agglomeration, dispersion and suspension stability may be influenced by external factors such as the flux of hydrogen ions (H+) in solution. Measurement of the Zeta potential of 0.1 wt.% CNC-EPTMAC suspensions over a wide range of pHs indicated that a pH range between 2 and 10 did not have a pronounced effect on the Zeta potential of the CNC-EPTMAC flocculant and did not decrease below 20 mV (Figure 2).

### 3.4. Chemical Properties

**Figure 3:**
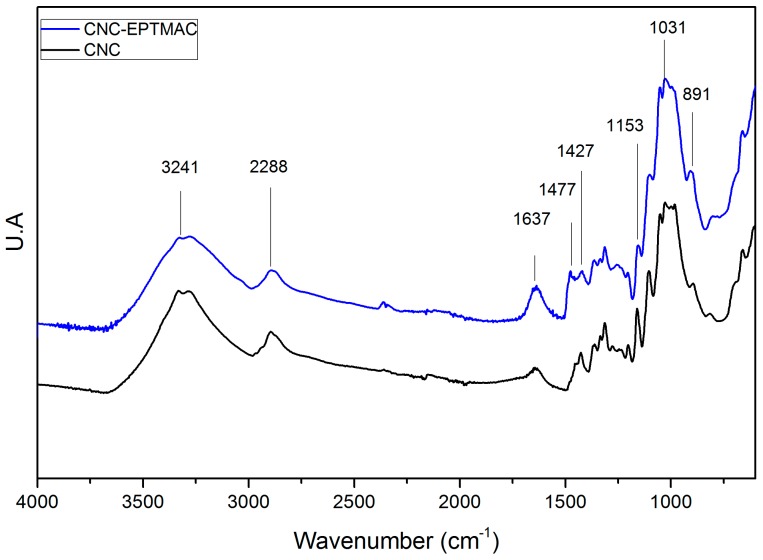
FT-IR absorbance spectra of CNC and CNC-EPTMAC.

The presence of the quaternary agent joined to the CNCs was confirmed by FT-IR (Figure 3). The infrared (IR) spectrum showed characteristic CNC signals: a strong band at 3241 cm^−1^, corresponding to the stretching of the hydroxyl group -OH; a band at 2288 cm^−1^, due to the symmetrical vibration of the C-H bond; a band at 1637 cm^−1^, originating mainly from humidity absorbed by CNCs; and the absorption band at 891 cm^−1^, which was assigned to the deformation of the carbon–hydrogen C–H bond of the glucosidic bond between the glucose units [27]. The absorption signal between 1030 cm^−1^ and 1153 cm^−1^ was due to the stretching of the carbon–oxygen bond (C–O), which is attributed to the ether’s main bonds. The IR-spectrum of CNC-EPTMAC prepared under conditions of 12 mol of CHPTAC per AGU for a 4-h reaction time at 25 ° C, clearly showed the quaternization of CNCs. The increase in the intensity of the main ether bands in the region between 1031 cm^−1^ and 1153 cm^−1^ is evidence of EPTMAC grafting on the surface of CNCs. Additionally, a prominent band was observed at 1477 cm^−1^ and 1427 cm^−1^ and was assigned to the CH_2_ bond and the bending of the methyl groups of the cationic substituent [28].

### 3.5. Crystalline Properties

**Figure 4:**
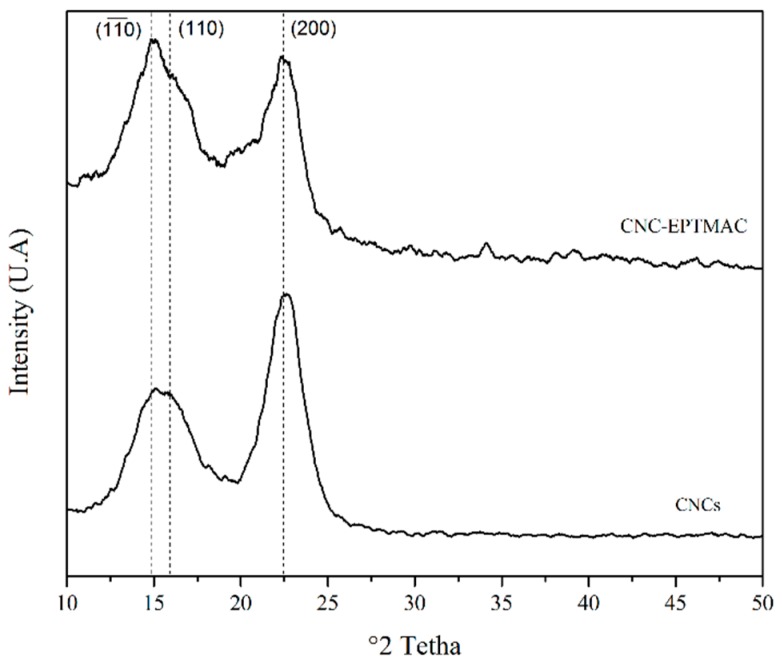
Crystallinity properties, XRD analysis.

The crystalline properties of unmodified CNCs and CNC-EPTMAC were analyzed by XRD (Figure 4). The typical peaks of Iβ cellulose located on the 2ϴ angle, were observed at 15.3° and 16.5° and were assigned to the planes (110) and (110), which are clearly solved for CNCs. The peak of the plane (200) was located at 22.6 ° and corresponded to the main crystalline region of cellulose I [35]. A Relative crystallinity index (RCI) of 83.6% was calculated for unmodified CNC, while an RCI of 74.7% was obtained after EPMAC-grafting. The decrease in RCI was due to the modification and suggests that another carbon, aside from C6, had been modified. However, the characteristic peaks of Iβ cellulose were maintained. The modification led to no significant changes in the signals of the CNCs; it can therefore be concluded that neither the exposure time to nor the molar ratio of the cationic agent changed the crystal structure of the CNCs, indicating that the modification takes place on the surface only. 

### 3.6. Thermal Properties

**Figure 5:**
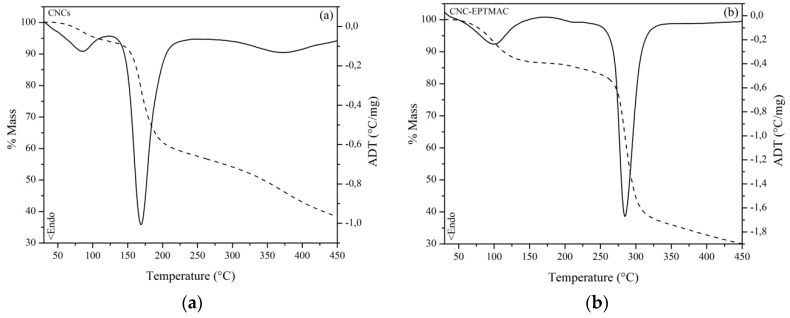
Thermogravimetric analysis (dotted line) and differential thermal analysis (unbroken line) of: (**a**) CNC and (**b**) CNC-EPTMAC.

Degradation of CNCs was observed in TGA outputs, and usually occurred in two steps (Figure 5a). The first step involved the removal of surface sulfate half ester groups; this began at a temperature of 150 °C, with an 8.41% loss of mass. Then, the cellulose was degraded in the temperature range of 250–450 °C, with a total mass loss of 42.46–61.63%. The 4.53% degradation at 100 °C was assigned to the loss of humidity, within the 2–5% range reported by others for CNCs at 100 °C [36,37]. The loss of sulfate groups at 150 °C, with a mass loss between 5-8% and degradation of cellulose between 250–500 °C, agreed with the experimental data. The CNCs modified with the cationic agent displayed variations in thermal properties (Figure 5b). Moreover, the CNC-EPTMAC thermogram showed a water loss of 7.05% at 100 °C, and two states of mass loss, the first taking place at 178 °C, with a mass loss of 13.57%, arising from the thermal decomposition of the quaternary ammonium groups grafted onto the surface of CNCs, and the second, beginning at 320 °C, with a mass loss of 61.56%, arising from the primary decomposition of the carbon skeleton [38]. 

Unmodified CNCs showed poor thermal stability; their T_di_ and T_dm_ values were lower as compared to those of CNC-EPTMAC. Additionally, a residual mass of 38.37% was measured for CNCs, while 30.03% was observed for CNC-EPTMAC at 450 °C (Table 2).

**Figure 6:**
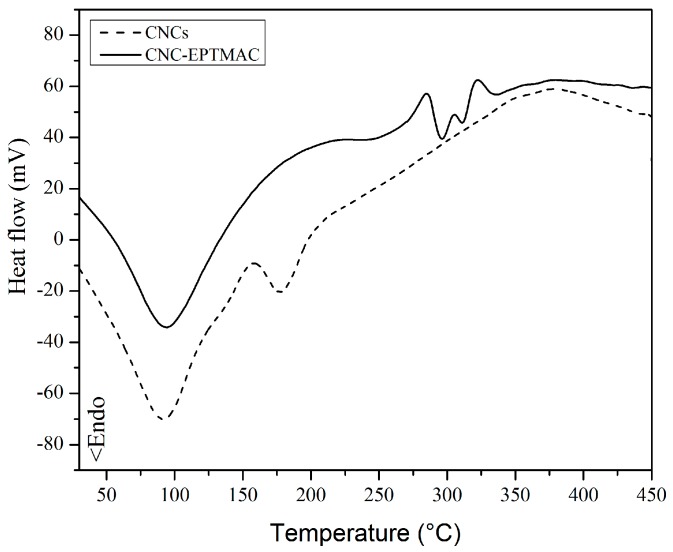
Differential scanning calorimetry analysis of CNCs (dotted line) and CNC-EPTMAC (unbroken line).

DSC analysis revealed two peaks (100 °C and 170 °C) for CNC (Figure 6). The first one corresponds to intramolecular humidity, while the second one suggests different decomposition mechanisms, possibly direct transitions from the solid to gas phase, catalyzed by surface sulfate groups. It has been reported that the activation energies of CNC degradation are significantly reduced upon the introduction of sulfate groups via hydrolysis with sulfuric acid [39]. Indeed, the thermal stability of the CNCs was compromised by the sulfate groups, while CNC-EPTMAC showed fusion endotherms at 100 °C, corresponding to intramolecular water molecules of humidity of the crystalline structure, and two more endotherms at 300 °C and 320 °C, corresponding to a cationic graft. 

### 3.7. Flocculation Treatment

**Figure 7:**
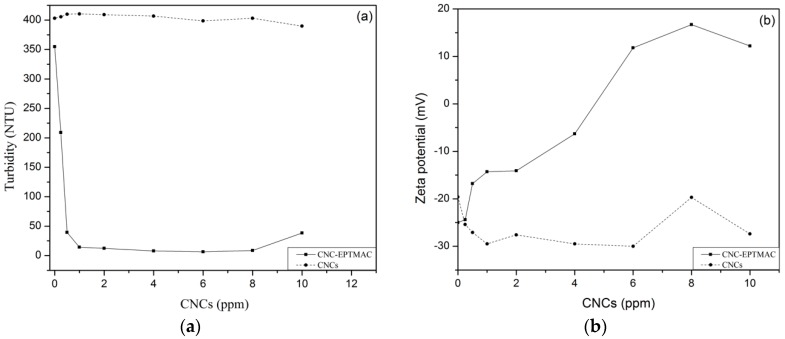
Flocculation test of modified versus unmodified CNC (**a**) turbidity and (**b**) Zeta potential change.

Flocculation potential was measured by turbidity reduction in a suspension of silica (SiO_2_, 0.25% wt.%), simulating wastewater. The flocculating effect of CNC-EPTMAC (0–10 ppm) was tested, with CNC samples serving as a negative control. While CNCs had no effect on the turbidity of the suspension, agglomeration was observed immediately after the addition of 0.25 ppm CNC-EPTMAC, (Figure 7a). The colloidal particle charges were destabilized in the presence of the cationic flocculant, which decreased the thickness of the electrical double layer of the particles, enabling van der Waals forces and the surface adsorption phenomenon to become dominant. As a result, an agglomerate formed, leading to the appearance of larger-weight floccules that later sedimented [40]. Figure 7b shows that 4-6 ppm CNC-EPTMAC resulted in the highest turbidity decrease and charge destabilization with Zeta potential values closer to the isoelectric point. Upon the addition of the control CNCs, the Zeta potential of the colloidal suspension did not change; namely, it neither destabilized nor neutralized charges, keeping the Zeta potential negative at all tested concentrations, with no decrease in turbidity. 

**Figure 8:**
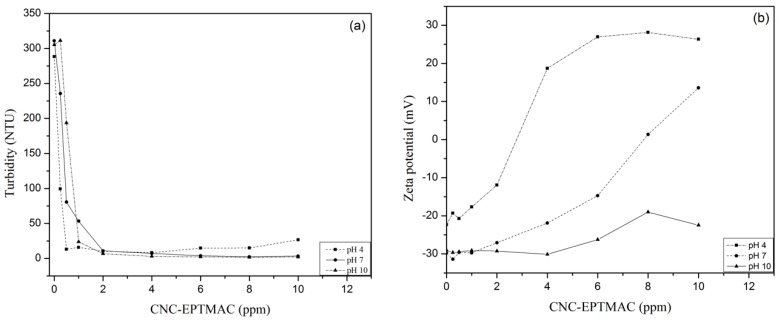
Effect of pH (acid pH 4, neutral pH 7 and alkaline pH 10) on flocculation. (**a**) Turbidity and (**b**) Zeta potential change.

The pH of the water matrix plays a key role in the decision to select a non-ionic or highly ionic flocculant. At acidic pHs, nonionic polymers show activity at relatively high concentrations. However, if a cationic polymer flocculent is used in an acidic pH, like in this study, lower concentrations will be required. This is due to the fact that at low pH, the hydrogen bonding sites available in the flocculant are reduced, which means that the cationic polymer will more effectively form bridge bonds and neutralize the charge [41]. For the acidic SiO_2_ suspensions, lower concentrations of flocculant were needed to reach an optimal effect. CNC-EPTMAC concentration requirements increased proportionally with pH, with the exception of pH 10, where the optimum dose of flocculant remained at 8 ppm (Figure 8). A change in pH can directly affect the surface charge of a colloid, leading to a change in Zeta potential, altering the stability of the system [42]. This work verified that the CNC-EPTMAC flocculant is effective at different concentrations in the pH range of 2 to 12.

**Figure 9:**
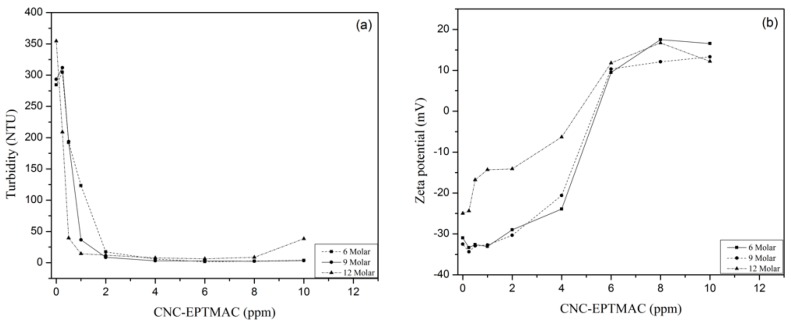
Effect of molar ratio on flocculation of SiO_2_ in the present of CNC-EPTMAC. (**a**) Turbidity and (**b**) Zeta potential change.

DS, i.e., the molar ratio of CNC-EPTMAC, is another key variable impacting the flocculation capacity. At a molar ratio of 12 mol of CHPTAC per AGU, less flocculant was required to remove a greater proportion of the turbidity of the SiO_2_ suspension, while the required concentration of flocculant increased as the molar ratios declined (Figure 9a). Likewise, Figure 9b shows that surface charge neutralization was achieved in the presence of a lower concentration of CNC-EPTMAC prepared with a molar ratio of 12 mol of CHPTAC per AGU as compared to CNC-EPTMAC prepared at a lower molar ratio, to achieve the optimal dose for the neutralization of the colloidal suspension of SiO_2_. This is due to the fact that the greater the molar ratio, the greater the DS, and therefore, there is a greater number of exposed cationic groups on the modification surface of the CNCs which can interact with the colloidal particles.

**Figure 10:**
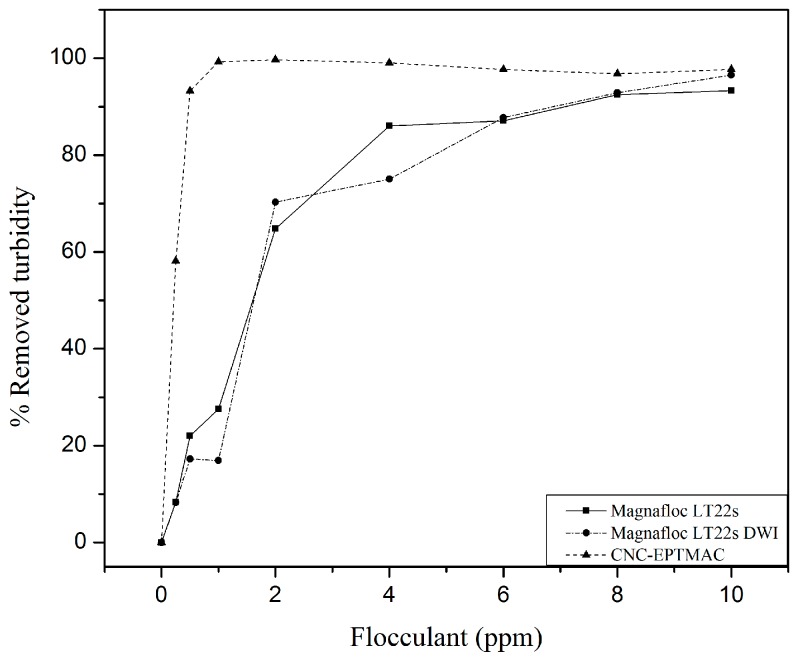
CNC-EPTMAC flocculation efficacy as compared to commercial flocculants.

Finally, a comparison of the effectiveness CNC-EPTMAC versus commercial flocculants with high cationic content and Zeta potential close to that of CNC-EPTMAC (Figure 10), showed that CNC-EPTMAC provided a greater turbidity reduction (99.7%) and had a lower optimal dose than commercial flocculants. This may be attributed to the fact that despite the equivalent Zeta potentials, the CNC-EPTMAC, due to its nanometric scale, has a greater surface area of contact with the particles in suspension. 

## 4. Conclusions

Our results show that CHPTAC can modify the surface of the CNCs without compromising their physical properties such as shape, dimensions and structure, and without intervening with their crystalline properties. Chemical characterization of the resulting CNC-EPTMAC showed that grafting of cationic quaternary ammonium groups on the surface of the CNCs resulted in a positively high Zeta potential, providing both stability in the dispersion and gained flocculant properties. Thermal properties were improved with the modification, increasing the decomposition temperature. In addition, CNC-EPTMAC demonstrated great potential as a flocculant in water treatment, offering significant stability in the range of 2 to 12 pH. Moreover, it effectively reduced turbidity by 99.7% at a concentration of only 2 ppm, showing superiority over and offering a natural and environmentally sustainable alternative to homologous commercial flocculants.

## Figures and Tables

**Table 1:** Conditions and results of the etherification reaction of CNC with CHPTAC.

Time (h)	Molar Ratio ^1^	Zeta Potential (mV)	Nitrogen (%)	DS ^2^	Size (d.nm)	PdI ^3^
4	6	33 ± 1.1	1.10	0.16	242.5	0.2
4	9	36.6 ± 0.2	1.33	0.21	235.7	0.2
4	12	40.4 ± 0.7	1.43	0.23	305.8	0.3
8	6	33.1 ± 0.1	1.11	0.17	247.2	0.2
8	9	37.6 ± 0.5	1.50	0.25	221.7	0.2
8	12	40.1 ± 1.3	1.45	0.24	234.7	0.2
24	6	33.8 ± 1.2	1.14	0.17	221.1	0.2
24	9	36.0 ± 1.0	1.90	0.36	203.5	0.2
24	12	38.8 ± 1.0	1.78	0.32	300.7	0.2
CNCs	0	−33 ± 2.1	0.00	0.00	113.7	0.4

^1^ Mol of CHPTAC per AGU, ^2^ Degree of substitution (DS) and ^3^ Polydispersity index (PdI)

**Table 2:** Decomposition temperature and residual mass of modified and unmodified CNC.

Sample	* ^1^ T_di_ (°C)	^2^ T_dm_ (°C)	^3^ T_df_ (°C)	Residual Mass (%)
CNCs	150	250	450	38.37
CNC-EPTMAC	178	320	450	30.03

* Decomposition temperature, ^1^ T_di_: initial, ^2^ T_dm_: maximum and ^3^ T_df_: final.
